# RNF7 knockdown inhibits prostate cancer tumorigenesis by inactivation of ERK1/2 pathway

**DOI:** 10.1038/srep43683

**Published:** 2017-03-02

**Authors:** Yangjiong Xiao, Yan Jiang, Hongmei Song, Tao Liang, Yonghui Li, Dongliang Yan, Qiang Fu, Zuowei Li

**Affiliations:** 1 Shanghai Sixth People's Hospital East, Shanghai University of Medicine & Health Sciences, Shanghai 201306, China; 2 Joint Research Center for Translational Medicine, East China Normal University and Shanghai Fengxian District Central Hospital, Southern Medical University, Nanfeng Road 6600, Shanghai 201499, China; 3 Shanghai Key Laboratory of Regulatory Biology, Institute of Biomedical Sciences and School of Life Sciences, East China Normal University, Shanghai 200241, China; 4 Department of Thoracic Surgery, Shanghai Pulmonary Hospital, Tongji University School of Medicine, Shanghai 200433, China

## Abstract

Development of castration resistance is a key contributor to mortality in patients with prostate cancer. High expression of RING finger protein 7 (RNF7) in cancer cells is known to play a key role in tumor progression. However, the role of RNF7 in prostate cancer progression is not well elucidated. In this study, we silenced RNF7 by shRNA interference in two castration resistant prostate cancer (CRPC) cell lines, DU145 and PC3. RNF7 knockdown attenuated proliferation and enhanced sensitivity of prostate cancer cells to cisplatin treatment. Invasive property of DU145 and PC3 cells was also attenuated by RNF7 silencing. The underlying mechanisms appear to be associated with accumulation of tumor suppressive proteins p21, p27 and NOXA, while inactivation of ERK1/2 by RNF7 knockdown. We demonstrated that RNF7 knockdown induced growth suppression of prostate cancer cells and inactivated ERK1/2 pathway, which suggested RNF7 might be a potential novel therapeutic target for CRPC.

Worldwide, an estimated 1.1 million men are diagnosed with prostate cancer (PC) every year^1^. Prostate cancer progression is amenable to repression at an early stage by androgen deprivation therapy (ADT). However, most patients with PC will eventually become castration resistant, which leads to its relapse. This feature makes prostate cancer the second cause of cancer death in men according to International Agency for Research on Cancer^2^,^3^,^4^. Hence, identification of molecules that are functionally associated with tumor initiation, progression, invasion and metastasis is a key research imperative in cancer therapeutics.

RNF7, also referred to as SAG (sensitive to apoptosis gene), ROC2 (regulator of cullins 2) or Rbx2 (RING-box 2), consists of 113 amino acids and has a molecular weight of 12.6 kDa. RNF7 was originally identified as a redox-inducible anti-oxidant protein^5^. It is a highly conserved protein and is extensively expressed in human skeletal muscles, heart and testis^5^. As a redox-inducible anti-oxidant protein, production of RNF7 can be induced by redox agents, which can protect cells from apoptosis caused by oxidation^5^. RNF7 is also a component of E3 ubiquitin ligases. When combining with other components to assemble E3 ubiquitin ligases, RNF7 has E3 ubiquitin ligase activity^6^. Although RNF7 is inducible by the transcription factor of activator protein-1 (AP-1), RNF7 inhibits tumor-promoting functionality of AP-1 by ubiquitinylation and degradation of c-Jun^7^. RNF7 has been shown to mediate ubiquitinylation of various cellular proteins such as, p21^8^, p27^9^,^10^, pro–caspase-3^11^, IκBα^12^,^13^, HIF-1α^14^, NOXA^15^ and NF1^16^. It is reported that RNF7 is over-expressed in many human cancers, such as carcinomas of lung, colon, stomach and liver^15^. Silencing of SAG expression by RNA interference was shown to inhibit proliferation of cancer cells (lung cancer cell line H1299, glioblastoma cell line U87, pancreatic carcinoma cell line PANC-1) *in vitro* and reverse radiation-resistance^15^. The mechanisms underlying this effect may include inactivation of NF-κB and mTOR pathway^8^ and/or accumulation of tumor suppressive proteins, such as NF1, DEPTOR, Procaspase-3, p21, p27, NOXA, and BIM^15^,^17^. As a component of E3 ubiquitin ligase, RNF7 may interact with massive cellular proteins. Further investigations are needed to reveal the novel functional aspects of RNF7 and their underlying mechanisms.

The role of RNF7 in prostate cancer, especially in castration resistant prostate cancer (CRPC), is not yet clear. In this study, we found that knockdown of RNF7 in two CRPC cell lines, DU145 and PC3, enhanced the sensitivity of these cells to cisplatin treatment. The underlying mechanisms were likely associated with increased cell apoptosis and inhibited ERK1/2 activity.

## Results

### RNF7 was efficiently silenced by RNA interference

**Figure 1:**
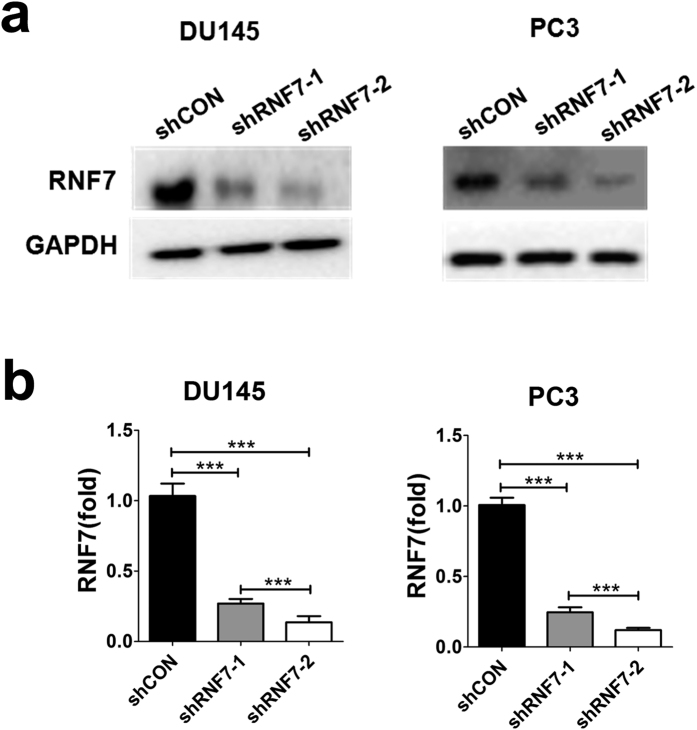
RNF7 expression was efficiently inhibited by shRNA interference. Prostate cancer cells were transfected with shRNF7-1, shRNF7-2 retrovirus or shCON negative control retrovirus. RNF7 expression was assessed by western blot (**a**), and confirmed on qRT-PCR (**b**). Results were representatives of three independent experiments. Data were mean ± SD. ***P < 0.001.

DU145 and PC3 cell lines were transfected with shRNF7 retroviruse (shRNF7-1 or shRNF7-2) or with negative control retrovirus (shCON). Protein and mRNA levels of RNF7 were determined by Western blot and qRT-PCR, respectively. RNF7 expression was significantly decreased after interference with shRNF7-1 or shRNF7-2 in both DU145 and PC3 cells (Fig. 1a).

To measure knockdown efficiency of RNF7, RNF7 mRNA expression level was determined by qRT-PCR. RNF7 transcription was statistically inhibited by shRNF7-1 or shRNF7-2 interference, *P* < 0.001 (Fig. 1b). RNF7 mRNA level in DU145 cells silenced by shRNF7-1 or shRNF7-2 compared to shCON-negative control was 26.2% ± 4.7% and 12.1% ± 6.4%, respectively. While the RNF7 mRNA level in PC3 cells silenced by shRNF7-1 or shRNF7-2 compared to shCON negative control was 24.9% ± 5.3% and 11.2% ± 2.4%, respectively. The silencing efficiency of shRNF7-2 was significantly greater than that of shRNF7-1, both in DU145 and PC3 cells, P < 0.001 (Fig. 1b). These data were consistent with reduced RNF7 protein expression detected by Western blot assay. Our results demonstrated that both shRNF7-1 and shRNF7-2 efficiently decreased RNF7 production.

### RNF7 Knockdown inhibited prostate cancer cell growth

**Figure 2:**
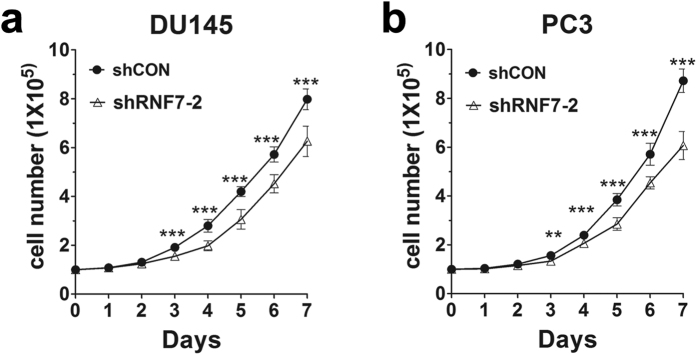
RNF7 knockdown inhibited cell proliferation. Cell numbers of DU145 (**a**) and PC3 (**b**) transfected with shCON or shRNF7-2 were monitored for 7 days. Data were mean ± SD. **P < 0.01; ***P < 0.001.

Rapid cell division is one of the properties of cancer cells. To determine the effect of RNF7 knockdown on prostate cancer cell proliferation, we seeded 1 × 10^5^ cells in 12 well plates and cultured them for 7 days. Cell numbers were recorded every day. Cell proliferation in the shRNF7-2 interfered group was significantly attenuated than that in the control (shCON) group from the third day onwards (*P* < 0.01) (Fig. 2a and b). On the 7^th^ day, the number of DU145 cells decreased to 78.4% by RNF7 interference (Fig. 2a), and 69.6% of PC3 cells (Fig. 2b). The results indicated significant attenuation of DU145 and PC3 cell proliferation after RNF7 knockdown.

### RNF7 Knockdown induced cell cycle arrest in prostate cancer cells

**Figure 3:**
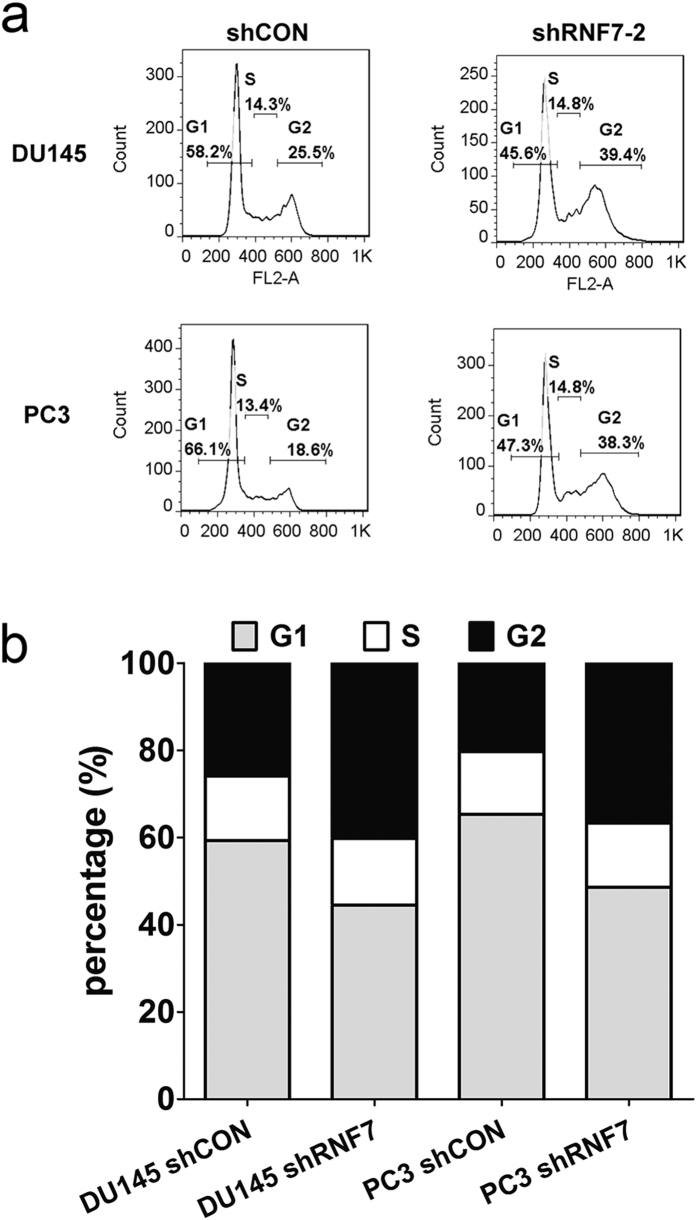
RNF7 knockdown induced cells arrested in G2 phase. Cell cycles were detected by PI staining using flow cytometry. (**a**) representative figures of cell cycle detection. Numbers were percentage of corresponding phases. (**b**) statistical data of cell cycle assay. G1: G1 phase, S: S phase, G2: G2 phase. shCON: cells transfected with empty vector as negative control, shRNF7: RNF7 knockdown cells.

To investigate the underling mechanism of growth suppression caused by RNF7 knockdown, cell cycle distribution of shRNF7 and shCON cells were measured by PI staining on flow cytometer. The mean percentage of negative control cells in G2 phase, in which RNF7 were normally expressed, were about 25.9% and 21.3% (Fig. 3a and b), respectively. However, mean percentage of G2 phase in RNF7 silenced DU145 and PC3 cells were about 41.2% and 38.7% (Fig. 3a and b), respectively. These results showed that RNF7 knockdown induced significantly G2 phase arrest in both DU145 and PC3 cells, P < 0.001.

### RNF7 knockdown accelerated prostate cancer cell apoptosis and cell death upon administration of chemotherapy

**Figure 4:**
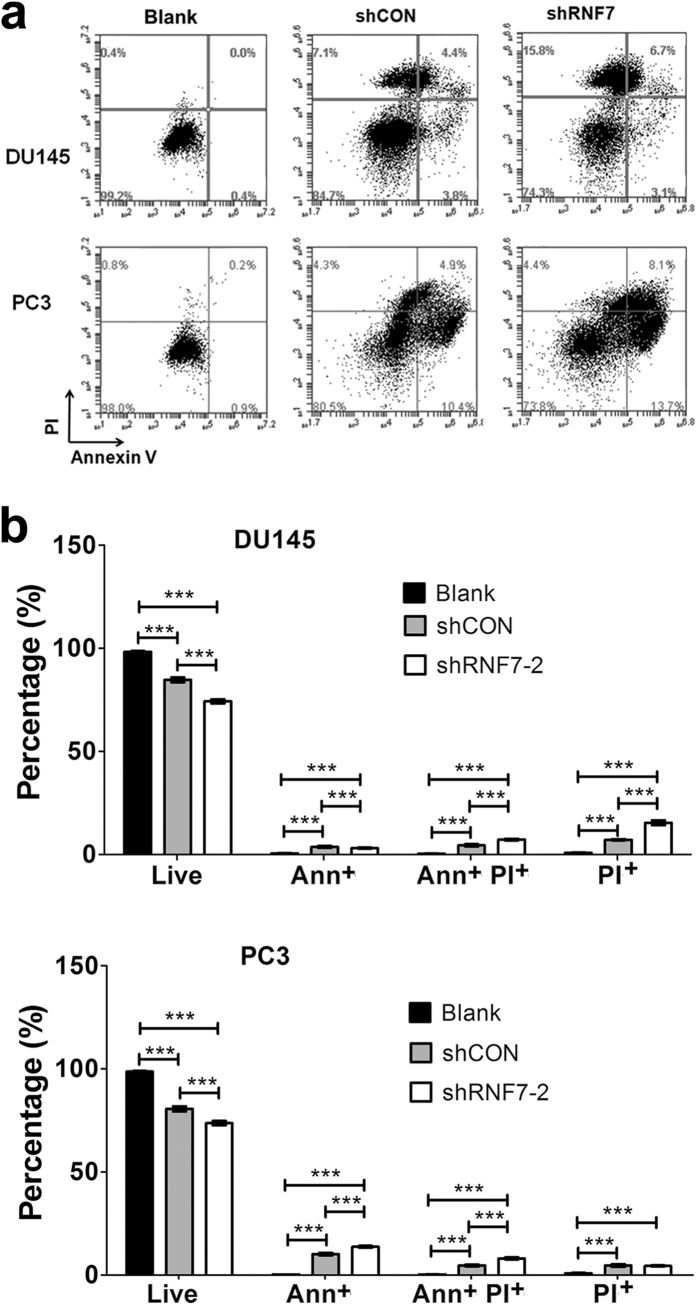
RNF7 knockdown induced higher percentage of DU145 and PC3 cell death to chemotherapeutic agent cisplatin treatment. Cells transfected with shRNF7-2 (shRNF7-2 group) or shCON (shCON group) were treated with cisplatin. Untreated cells served as negative controls. Cell apoptosis was detected by Annexin V and PI staining. (**a**) Representative results of Annexin V and PI staining; (**b**) Statistical analysis of cell apoptosis. Live: Annexin V and PI double negative cells. Ann^+^: Annexin V positive but PI negative cells. Ann^+^ PI^+^: Annexin V and PI double positive cells. PI^+^: PI positive but Annexin V negative cells. Data were mean ± SD. ***P < 0.001.

To investigate whether RNF7 knockdown had synergetic effect with chemotherapy on prostate cancer treatment, DU145 and PC3 cells transfected with shRNF7-2 or shCON were treated with cisplatin. Apoptosis cells were positively stained with Annexin V or double positively stained with Annexin V and PI, while dead cells were only PI positive (Fig. 4a). In DU145 cells, cisplatin combined with shRNF7-2 interference induced significantly higher apoptosis or necrosis indicated by Annexin V and/or PI staining positive (25.7%) as compared to that induced by cisplatin alone (15.3%) (*P* < 0.0001) (Fig. 4b). Also, cisplatin induced significantly higher cell apoptosis or necrosis (26.3%) in shRNF7-2 silenced PC3 cells compared to shCON control group (19.4%), *P* < 0.0001 (Fig. 4b). So, RNF7 silencing enhanced the sensitivity of prostate cancer cell lines to cisplatin.

### RNF7 Knockdown inhibited prostate cancer cell invasion

**Figure 5:**
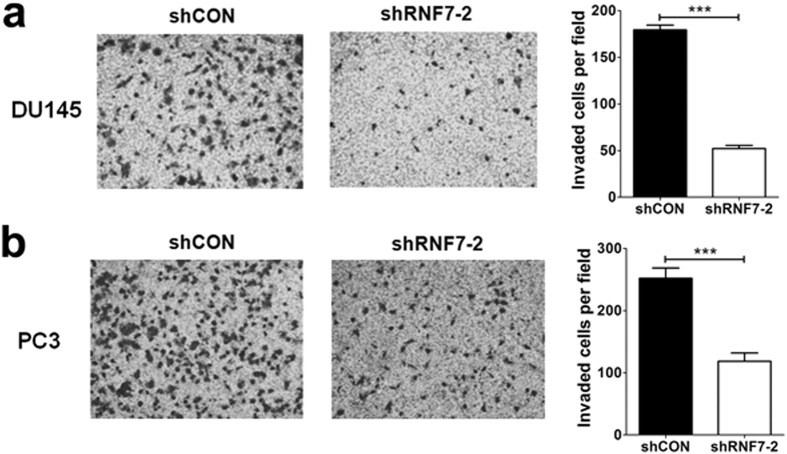
RNF7 knockdown decreased prostate cancer cell invasion. Invaded cells of shRNF7-2 interfered DU145 cells (**a**) and PC3 cells (**b**) were significantly lower than that of shCON negative controls. Representative figures and statistical data were showed. Data were mean ± SD. ***P < 0.001.

Cell invasion plays a critical role in cancer relapse and causes death. The role of RNF7 in DU145 and PC3 cell invasion were analyzed by invasion assay. Our results showed that the invasive ability reduced significantly after RNF7 silencing in DU145 (Fig. 5a) and PC3 (Fig. 5b) cells, *P* < 0.0001.

### RNF7 Knockdown inhibited prostate cancer cell colonization and tumorigenesis

**Figure 6:**
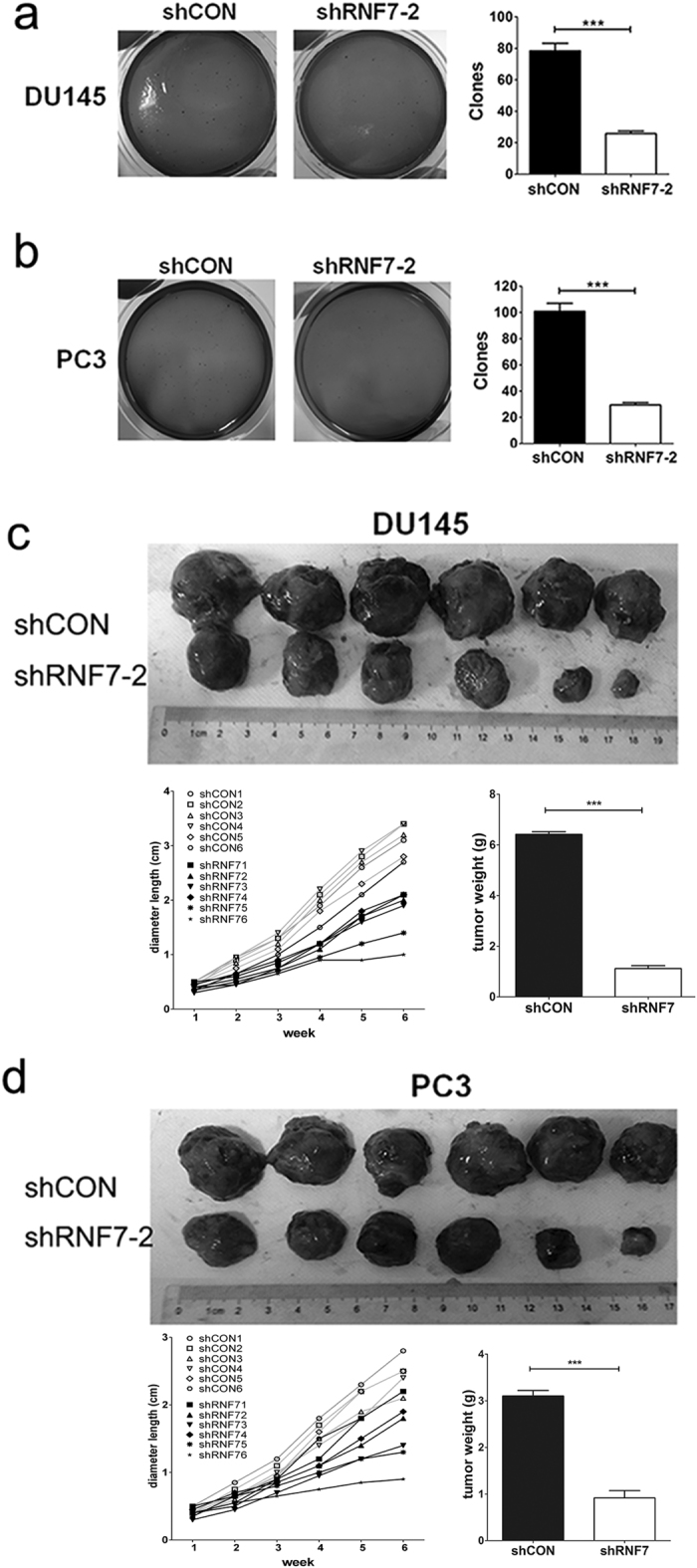
RNF7 knockdown inhibited prostate tumorigenesis. Colonies formed by DU145 (**a**) and PC3 (**b**) shRNA interfered cells (shRNF7-2 group) were significantly lower than those formed by negative controls (shCON group). DU145 (**c**) or PC3 cells (**d**) (both shNRF7 interfered and shCON negative controls) were implanted subcutaneously into the back of nude mice. Each group contained 6 nude mice. Mice were sacrificed six weeks later. Xenografts were collected and their weights were measured. Representative photos and statistical data were showed. Data were mean ± SD. ***P < 0.001.

Colony formation indicates the ability of tumorigenesis. Clonogenic survival assay was performed to detect the role of RNF7 in prostate cancer cell colony. As inspected, DU145 (Fig. 6a left panel) and PC3 (Fig. 6b left panel) cells exhibited higher colony forming ability. However, this ability was significantly compromised by RNF7 interference, *P* < 0.0001 (Fig. 6a and b, middle and right panels). The size and weight of tumors developed from DU145 (Fig. 6c) or PC3 (Fig. 6d) cells in nude mice were significantly decreased by RNF7 silencing, *P* < 0.0001. These data demonstrated that RNF7 knockdown inhibited tumorigenesis in prostate cancer cells.

### RNF7 Knockdown induced accumulation of p21, p27 and NOXA, and inhibition of ERK1/2 activity

**Figure 7:**
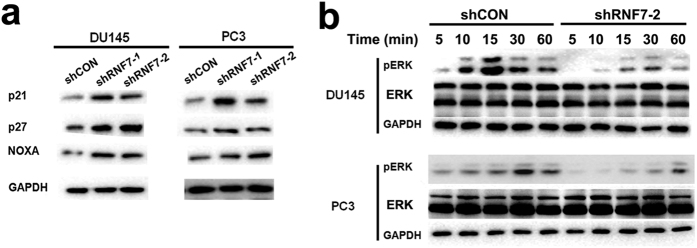
RNF7 knockdown induced cell growth inhibition. (**a**) Expression of pro-apoptosis protein NOXA and tumor suppressor proteins p21 and p27 was detected by western blot assay. shRNF7-1 or shRNF7-2: cells interfered by shRNF7-1 or shRNF7-2 retrovirus. (**b**) Both shRNF7-2 interfered (shRNF7-2 group) or the negative control (shCON group) prostate cells were stimulated with EGF for 5 to 60 minutes, and expression or phosphorylation of ERK was detected by Western blot assay.

In both DU145 and PC3 cells, the expression of pro-apoptosis protein NOXA and tumor suppressor proteins p21 and p27 was highly up-regulated by RNF7 silencing (Fig. 7a). ERK plays a critical role in tumor cell survival and proliferation. And phosphorylation indicates activation of ERK. To investigate the effects of RNF7 knockdown on ERK activation, prostate cancer cells were activated by epidermal growth factor (EGF) for 5 to 60 minutes and ERK phosphorylation was detected by Western blot. Our study showed that it was the activity not the expression of ERK that was inhibited by RNF7 knockdown in DU145 and PC3 cells (Fig. 7b). These data demonstrated that RNF7 knockdown up-regulated expression of pro-apoptosis and/or tumor suppressor proteins, while inhibited the activity of ERK pathway.

**Figure 8:**
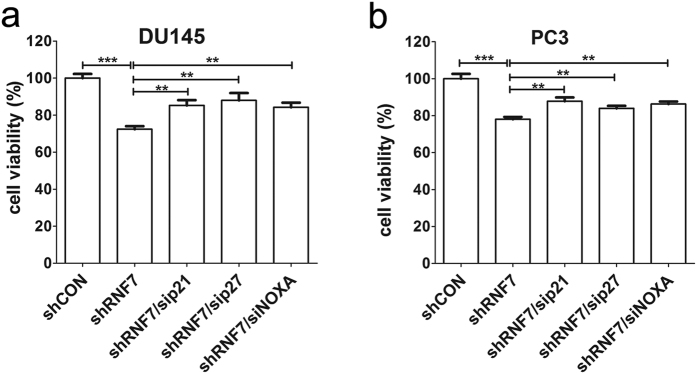
Simultaneous knockdown of P21, P27 or NOXA with RNF7 partially reversed growth suppression caused by RNF7 interference. RNF7 silenced (shRNF7 group) DU145 (**a**) or PC3 (**b**) cells were interfered by siRNAs targeting p21, p27 or NOXA, respectively. Cell viability was measured by MTT assay 4 days after siRNA interference. Viability of DU145 or PC3 cells normally expressed RNF7 (shCON control group) was set as 100 percent. sip21: cells interfered by siRNA targeting p21. sip27: cells interfered by siRNA targeting p27. siNOXA: cells interfered by siRNA targeting NOXA. Statistical data were mean ± SD. **P < 0.01, ***P < 0.001.

To verify that proliferation suppression was associated with accumulation of p21, p27, and NOXA in RNF7 silenced prostate cancer cell, p21, p27, or NOXA was silenced by siRNA in RNF7 knockdown DU145 and PC3 cells. Four days after siRNA interference, cell proliferation was measured by MTT assay. Our results showed that cell proliferation increased significantly by silencing of p21, p27, or NOXA in both RNF7 knockdown DU145 (Fig. 8a) and PC3 cells (Fig. 8b). These results showed that the suppressive effect of RNF7 knockdown on cell growth was likely attributable to accumulation of P21, P27 and NOXA.

In conclusion, our study demonstrated that RNF7 knockdown could suppress proliferation and inhibit tumor formation of prostate cancer cells. The underlying mechanisms were associated with accumulation of tumor suppressive proteins p21, p27 and NOXA, and inactivation of ERK1/2 pathway.

## Discussion

Prostate cancer is one of the leading causes of death in males worldwide. While most prostate cancer patients respond to ADT initially, a vast proportion of them will eventually relapse due to castration resistance^2^,^3^,^4^. Hence, development of treatment regimens that reverse castration resistance is a key priority in CRPC research.

Chemotherapy is one of the main therapeutic modalities for CRPC. It was reported that docetaxel could prolong survival time 2 to 3 months^18^. Other chemotherapeutic agents used for improving treatment efficacy either singly or in combination with docetaxel include, cisplatin^19^,^20^, estramustine^21^, 153Sm-lexidronam^22^, prednisolone^23^, mitoxantrone^24^,^25^, epirubicin^26^, bortezomib^27^, zoledronic acid^28^, capecitabine^29^, and vinorelbine^30^. Other regimens were used or studied in clinical trials include, combination of estramustine phosphate, ifosfamide and cisplatin^20^; prednisone plus cabazitaxel and prednisone plus mitoxantrone^25^; epirubicin and cisplatin^31^; and cisplatin plus prednisone^32^. Although these regimens enhanced the 50% survival time up to 15–20 months, more efforts are needed for improving the treatment efficacies. It was reported that cisplatin induced reactive oxygen species (ROS) in prostate cancer cells. And the production of ROS in hormone-sensitive LNCap cells was significantly higher than that of CRPC DU145 and PC3 cells^33^. Increasing the ROS production in CRPC cells may improve efficiency of chemotherapy especially cisplatin treatment.

RNF7 is highly expressed in various human cancers, such as lung, liver and stomach cancers^15^. Also we found high expression level of RNF7 in prostate DU145 and PC3 cells. RNF7 was proved to be an antioxidant by Yi Sun and his colleagues^5^. Then we hypothesized that silencing of RNF7 might have a synergistic effect with cisplatin chemotherapy. So we sought to investigate the effect of RNF7 knockdown on sensitivity of DU145 and PC3 cells to cisplatin treatment. Retrovirus based shRNA were used to silence RNF7 expression. The knockdown efficiency was comparable to that reported in previous studies^7^,^8^. RNF7 knockdown significantly inhibited DU145 and PC3 cell proliferation starting from the third day, which may be associated with RNF7 knockdown induced cell cycle arrest in G2 phase. Consistent with our study, Tan *et al*. recently showed that RNF7 knockdown by lentivirus-based siRNA significantly decreased prostate cancer cell proliferation^34^. We also investigated the effect of RNF7 knockdown on CRPC cell sensitivity to cisplatin. RNF7 knockdown combined with cisplatin chemotherapy induced significantly higher percentage of Annexin V positively stained apoptosis cells as compared to that observed with cisplatin treatment only. The underlying mechanism might be associated with increased expression of pro-apoptosis protein NOXA in RNF7 silenced cells. We further investigated the effect of RNF7 silencing on cancer cell invasion. RNF7 knockdown significantly inhibited invasive properties of DU145 and PC3 cells. Consistent with our study, Tan *et al*. also showed that RNF7 knockdown significantly inhibited prostate cancer cell migration and clone formation *in vitro*^34^. Based on the above results, we hypothesized that RNF7 silencing might counteract CRPC tumorigenesis. Our study showed that clonogenic forming ability of DU145 and PC3 *in vitro*, and its tumor formation ability in nude mice were significantly decreased by RNF7 interference. These results were consistent with increased expression of tumor suppressor proteins p21 and p27. Similar findings have been reported from a previous study on lung cancer cells^8^. We also sought to figure out the potential signal transduction pathway involved in RNF7 knockdown mediated cell growth inhibition. While Tan *et al*. showed that RNF7 knockdown decreased AKT activation^34^, we observed inactivation of ERK1/2 activity by RNF7 silencing. Multiple signaling pathways might be involved in the inhibition of cell growth and tumorigenesis mediated by RNF7 knockdown. To address further whether ERK1/2 inactivation is associated with inhibition of RAS activity, we detected RAS activity of shRNF7 and shCON cells, and we did not observe RAS inactivation caused by RNF7 interference (data not shown), indicating ERK1/2 inactivation by RNF7 knockdown might not due to RAS inactivation by potential NF1 accumulation. More investigations are needed to uncover the underlying mechanisms. To our knowledge, this is the first study to demonstrate that ERK1/2 activation is regulated by RNF7 in cancer cells.

In conclusion, our study demonstrated that RNF7 knockdown inhibited prostate cancer cell proliferation and tumorigenesis, suggesting that RNF7 might be a promising target for CRPC treatment. The underlying mechanisms might be associated with accumulation of tumor suppressive proteins p21, p27 and NOXA, and inactivation of ERK1/2 activity.

## Materials and Methods

### Cell lines

Human prostate cancer cell lines DU145 and PC3, and HEK293T were purchased from American Type Culture Collection (ATCC) and cultured in high glucose DMEM medium (GIBCO, Grand Island, USA) containing 10% fetal bovine serum (FBS) (GIBCO), 100 U/ml penicillin (GIBCO) and 100 μg/ml streptomycin (GIBCO).

### shRNA constructs of RNF7

The following two oligos were used for construction of shRNAs against RNF7: shRNF7–1 oligo: TGCTGTTGACAGTGAGCGACCGGCTAATTTTTGTATTTTTTAGTGAAGCCACAGATGTAAAAAATACAAAAATTAGCCGGGTGCCTACTGCCTCGGA. shRNF7-2 oligo TGCTGTTGACAGTGAGCGCCACCTTTATAATTTACCCATTTAGTGAAGCCACAGATGTAAATGGGTAAATTATAAAGGTGATGCCTACTGCCTCGGA. These two oligos were amplified using the following primer pair miR30 XhoI (5′-CAGAAGGCTCGAGAAGGTATATTGCTGTTGACAGTGAGCG-3′) and miR30 EcoRI (5′-CTAAAGTAGCCCCTTGAATTCCGAGGCAGTAGGCA-3′). Thus, each of the RNF7 targeting sequences shRNF7-1 and shRNF7-2 contained miR30 sequences and enzyme sites of XhoI and EcoRI. shRNF7-1 and shRNF7-2 were subcloned into LMP vector by polymerase chain reaction (PCR) and confirmed by DNA sequencing.

### Packaging of Retroviruses in HEK293T cells and transfection of prostate cancer cell lines

Recombinant LMP vectors containing shRNF7-1 or shRNF7-2 combined with pCL10A1 retrovirus packaging vector were transfected into HEK293T cells using lipofectamine 2000 (Life Technologies, Carlsbad, CA, USA), according to manufacturer’s instructions. After 48 h incubation, retroviruses containing shRNF7-1 or shRNF7-2 were collected from culture supernatant. pCL10A1 retrovirus and LMP vector without shRNF7 sequences served as the negative control (shCON).

Prostate cancer cells were transfected with retroviruses in the presence of 10 μg/ml polybrene and selected by 1 μg/ml puromycin. Total RNA of prostate cancer cells was extracted using Trizol reagent (Invitrogen, Madison, WI, USA). To determine the efficiency of RNA interference, RNF7 mRNA levels were assessed by quantitative RT-PCR (qRT-PCR), and RNF7 protein levels were assessed by Western blot analysis.

### qRT-PCR analysis

Total RNA was reverse transcribed into cDNA using Superscript III Reverse transcriptase kit (Life Technologies). mRNA levels were determined using SYBR^®^ Green Real-time PCR master mix (Takara, Shiga, Japan) on 7900HT qRT-PCR machine (Applied Biosystems, Foster City, CA, USA). The following pair of primers of RNF7 were used for amplification: 5′-TGGAAGACGGAGAGGAAACCT-3′, 5′-TCCCCAGACCACAACACAGT-3′. Glyceraldehyde-3-phosphate dehydrogenase (GAPDH) gene was used as an internal control. The primers of GAPDH were 5′-GACAGTCAGCCGCATCTTCT-3′ and 5′-TTAAAAGCAGCCCTGGTGAC-3′^35^. Relative mRNA levels were analyzed as the ratio of RNF7 to GAPDH using ΔΔCt method.

### Cell proliferation assay

DU145 and PC3cell lines transfected with or without RNF7 shRNA were seeded into 12 well plates at a concentration of 2 × 10^5^ cells/ml with a total volume of 0.5 ml/well. Cell numbers were recorded every day using Bio-Rad cell counter (Bio-Rad Laboratories, Hercules, CA, USA). Growth curves of cells (number of cells versus time) were plotted for up to seven days. RNF7 knockdown DU145 and PC3cells interfered by siRNA targeting p21 (SASI_Hs01_00025255, sigma), p27 (SASI_Hs01_00113637) or NOXA (SASI_Hs01_00136187) were seeded to 96 wells with 2 × 10^4^/ml in a total volumn of 200 μl. Cell viability was detected by Methylthiazolyldiphenyl-tetrazolium bromide (MTT, M5655, sigma) after 4 days culturing.

### Cell cycle assay

Both RNF7 knockdown and the negative control DU145 and PC3 cells were harvested and fixed by 70% ethanol at 4 °C for 24 h. Cells were incubated with 50 μg/ml propidium iodide (PI) containing 400 U/ml RNase A for 30 min at room temperature in the dark. Cell cycle distribution was measured by FACSCalibur flow cytometer.

### Apoptosis assay

DU145 and PC3 cells transfected with or without RNF7 shRNA were seeded into 24-well plates in 0.5 ml culture medium (2 × 10^5^ cells/ml). Cells were treated with or without 10 μg/ml cisplatin (P4394, Sigma) for 24 h. Cells were harvested by centrifugation and stained with Annexin V and 1 μl propidium iodide (PI) (V13242, ThemoFisher Scientific) according to the manufacturer’s instruction. Cells were detected by FACSCalibur C6 (BD Biosciences, San Jose, CA, USA) and analyzed using FlowJo software (FlowJo LLC, Ashland, OR, USA).

### Cell invasion assay

Cell invasion was assessed using 24-well Boyden chamber plates which contained polycarbonate membrane filter inserts with 8-μm pore size (Costar Group, Washington, DC, USA). Diluted Matrigel (BD Biosciences) was added to the interior of the transwell inserts for mimicking of basement membrane. The upper chambers were seeded with 1 × 10^5^ cells and the lower chambers were filled with 1 ml DMEM containing 10% FBS, and cells were cultured for 24 h. Subsequent to removal of non-migrating cells present on the upper chamber surface, invaded cells at the bottom of the membrane were fixed with methanol and stained with crystal violet. Invaded cell numbers were recorded under microscope.

### Clonogenic survival assay

Ten thousand prostate cancer cells transfected with shRNF7 retrovirus or negative controls were resuspended in DMEM medium containing 10% FBS and 0.3% agarose. Cells were plated onto a solidified bottom layer in DMEM medium containing 10% FBS and 0.5% agarose in 60-mm culture dishes. The colonies that formed at day14 were recorded following fixation with methanol, and stained with 0.05% methylene blue.

### Tumor formation assays in nude mice

One million DU145 or PC3 cells transfected with or without RNF7 shRNA in 100 μl phosphate buffer saline (PBS) were injected subcutaneously (s.c.) into the flanks of 6-week-old female nude mice. Tumor growth was monitored for 6 weeks. Tumors were weighted and imaged after surgical removal.

### Western blot analysis

Cells were lysed using RIPA lysis buffer (Life Technologies) and protein amount were determined by BCA protein assay kit (ThermoFisher Scientific). Equal amount (10 μg) of proteins were loaded and resolved onto 8–12% SDS-PAGE gel and blotted onto polyvinylidene fluoride (PVDF) membrane by electrophoresis. Following antibodies were used to detect the corresponding proteins: rabbit anti-RNF7 antibody (Ab181986, Abcam, Cambridge, MA, USA); rabbit anti-p21 antibody (#2947, CST, cell signaling technology, Danvers, MA, USA); rabbit anti-p27 antibody (#3686, CST); rabbit anti-NOXA antibody (#14766, CST); rabbit anti-ERK1/2 antibody (#4695, CST); rabbit anti-pERK1/2 antibody (#4370, CST); rabbit anti-GAPDH antibody (#5174, CST); HRP conjugated goat anti-rabbit IgG (#7074, CST). HRP Chemiluminescent substrate (ECL) kit (# 32106, ThermoFisher Scientific) was used for color development.

### Statistical analysis

Data were presented as mean ± Standard Deviation (SD). All experiments were repeated at least three times independently with three or more replicates. Statistical differences were determined by GraphPad Prism 5.0 software (GraphPad Software Inc., CA, USA). Gaussian distribution data were analyzed by two-tailed Student’s *t* test, while non-Gaussian distribution data by Mann-Whitney of nonparametric test.

### Ethic statements

All animal procedures were performed in accordance with Guidelines of Shanghai JiaoTong University School of Medicine, and approved by the Animal Care and Use committee of Shanghai Jiaotong University School of Medicine.

## Additional Information

**How to cite this article**: Xiao, Y. *et al*. RNF7 knockdown inhibits prostate cancer tumorigenesis by inactivation of ERK1/2 pathway. *Sci. Rep.* **7**, 43683; doi: 10.1038/srep43683 (2017).

**Publisher's note:** Springer Nature remains neutral with regard to jurisdictional claims in published maps and institutional affiliations.
